# Patient-centred outcomes of a rheumatology podiatry service for people with foot-specific symptoms: protocol for a randomised feasibility trial

**DOI:** 10.1007/s00296-025-06039-3

**Published:** 2026-02-10

**Authors:** Glen A. Whittaker, Claire E. Owen, Anna S. Antony, Alicia M. James, Kylie Latu, Hylton B. Menz

**Affiliations:** 1 Discipline of Podiatry, School of Allied Health, Human Services and Sport, La Trobe University, Melbourne, Victoria 3086 Australia; 2 Austin Health, Heidelberg, Victoria 3805 Australia; 3 Monash Health, Clayton, Victoria 3168 Australia; 4 College of Sport, Health and Engineering, Victoria University, Melbourne, Victoria 3011 Australia; 5 Department of Medicine, The University of Melbourne, Parkville, Victoria 3010 Australia

**Keywords:** Rheumatology, Podiatry, Foot, Feasibility studies, Patient-centered care

## Abstract

People with rheumatological conditions often experience debilitating foot-related symptoms requiring expert multidisciplinary care. However, in Australia there is a glaring gap in the provision of universally accessible, publicly-funded podiatry services. This study aims to assess the feasibility of a randomised trial evaluating a publicly-funded podiatry service for people with foot-specific symptoms related to rheumatological conditions. A pragmatic, participant-blind, parallel-group, randomised feasibility trial will compare effectiveness of a podiatry service with usual care. Consumer interviews will inform the design of the feasibility trial. Thirty adults with a rheumatological condition and foot specific symptoms will be recruited from an outpatient rheumatology clinic and randomised to receive access to a podiatry service or a control group who will receive usual care. Outcome measures will be obtained at baseline, week 6 and week 12. Feasibility will be evaluated using a framework that includes 10 important domains of uncertainty in pragmatic feasibility trials. Secondary outcomes (such as Goal Attainment Scaling) will also be collected and effect sizes of between group differences calculated to signal efficacy. The study will explore the feasibility of conducting a fully powered randomised trial of the effectiveness of a podiatry service for people with a rheumatological condition and foot-specific symptoms. In addition, the trial will determine the feasibility of Goal Attainment Scaling as a primary outcome measure for this population.

Trial registration.

The trial is registered with the Australian and New Zealand Clinical Trial Registry (ANZCTRN12625001000493 on the 9th of September 2025).

## Introduction

People living with rheumatological conditions frequently endure debilitating foot-related symptoms. Studies indicate that up to 90% of adults with rheumatoid arthritis suffer from foot pain, significantly impacting their daily activities and overall well-being [1]. The presence of foot and ankle symptoms correlates with increased disease activity and disability, leading to a diminished quality of life [2]. Moreover, foot ulceration is a prevalent and serious complication among people with chronic rheumatoid arthritis, affecting up to 4–10% of patients [3, 4]. Foot ulceration is not exclusive to rheumatoid arthritis, and is also observed in systemic lupus erythematosus [5], and systemic sclerosis and small vessel vasculitis [6]. Systemic lupus erythematosus, a connective tissue disease, is particularly notorious for affecting the feet, resulting in an array of vascular, dermatological, and musculoskeletal issues [7]. Despite advancements in treatment and the introduction of novel disease-modifying antirheumatic drugs, these foot-specific symptoms may persist, underscoring the need for targeted research and improved management strategies to enhance the quality of life [8, 9].

Rheumatological conditions are complex, and require patient-centred, multidisciplinary approaches that address the many ways these conditions affect health and wellbeing. Patient-centred multidisciplinary team-based care is recommended in the recent Australian Rheumatoid Arthritis Clinical Care Standard [10] and Juvenile Idiopathic Arthritis Standard of Care [11], and is advocated for by Arthritis Australia [12]. However, the reality is that the provision of such care is contingent upon the availability of a diverse team of healthcare professionals. A glaring gap in this team is the provision of podiatric care, which is somewhat of a ‘black hole’ [13]. Previous research has found a stark absence of podiatrists within public rheumatology departments across Australia [14], and there is a significant disconnect in the integration of podiatry into existing care teams [15]. This is despite podiatry-led management of foot symptoms being effective for people with a range of rheumatological conditions [16]. The need for specialised podiatry care is clear and pressing, yet the current system fails to meet this need, which underscores the challenges faced in adhering to the minimum standards of multidisciplinary care.

Recognising the critical role of patient-centred multidisciplinary team care in managing foot-related symptoms for individuals with rheumatological conditions, the need for universally accessible, publicly-funded podiatry services is clear. At present, access to podiatric care is largely restricted to the private sector, creating a significant barrier to equitable health provision. This study aims to assess the feasibility of implementing a pragmatic randomised trial to evaluate the effectiveness and cost-effectiveness of a publicly-funded podiatry service tailored for people suffering from foot-related symptoms due to rheumatological conditions. Furthermore, the study will explore patient-centred goal-setting as an outcome measure and obtain data to inform sample size calculations for a randomised trial.

## Methods

This will be a pragmatic, participant-blind, parallel-group, randomised feasibility trial comparing access to a podiatry service with usual care. The trial is registered with the Australian and New Zealand Clinical Trial Registry (ANZCTRN12625001000493) and has been developed in accordance with the SPIRIT 2025 statement [17], the CONSORT 2010 statement extension to randomised pilot and feasibility trials [18], and the TIDieR checklist for reporting of interventions [19]. Ethics approval has been obtained from the La Trobe University Human Ethics Committee (Approval number HEC25095 on the 20th of May 2025) and written informed consent will be obtained from all participants during the baseline assessment.

The randomisation module in REDCap (Research Electronic Data Capture, Vanderbilt University, USA) will be used to randomise participants. Participants will be allocated on a 1 to 1 ratio with blocking in groups of 4 and 6, and stratification based on sex at birth to ensure a balance of participant sex between the groups. Allocation will be concealed from all researchers until after eligibility screening. Participants will be randomised to their intervention after completing consent at the baseline assessment.

Given this is a feasibility trial, only participants will be blind to their allocation. Both groups will be blind to group allocation through limited disclosure. All participants will be informed that two different types of treatment are being compared, however only the intervention group will be informed about the additional access to podiatry treatment.

### Study groups

The podiatry service will operate from the La Trobe University Health Sciences Clinic in Melbourne, Australia. Given this is a pragmatic trial, participant needs will be triaged during the baseline assessment, which will determine the types of interventions provided by the podiatry service. Based on previous research [20], it is anticipated that treatment will involve broad categories of:

1. dermatological care (nail and callus care, education);
2. musculoskeletal care (managing pain, deformity, and improve function using: (i) medication in conjunction with medical team, (ii) strengthening exercise and stretches, (iv) foot orthoses, (v) in-shoe padding, (vi) toe splints, and (vii) education);
3. neurovascular care (assessment, education, management where appropriate, and referral if needed);
4. foot ulcer management (pressure offloading, wound care and dressing, and referral where appropriate);
5. footwear education and modification.

Treatment will be provided by a podiatrist with over 10 years of experience in clinical practice, who is endorsed to prescribe scheduled medicines, has undertaken further training in rheumatological conditions, and plays an active role in the Australian Rheumatology Association. For participants who require foot orthoses, Formthotics™ (Foot Science International, Christchurch, New Zealand) full length prefabricated foot orthoses will be provided. These are manufactured from a dual-density polyethylene closed cell foam that has a firm-density bottom layer (Shore-A durometer 50), and a soft-density top layer (Shore-A durometer 25). Participants will be provided the foot orthoses in accordance with the manufacturer’s instructions by selecting an appropriate size, heating the foot orthoses using a specifically designed machine from Foot Science International, placing them within the participants footwear, and asking participants to stand to allow the foot orthoses to mould to the contour of the foot. Adjustments to the foot orthoses will be made to ensure the participants agree they are comfortable. Participants who require footwear will be provided with a $250 discount from the cost of new footwear. The footwear will be agreed with the podiatrist and participants will be required to attend pre-arranged footwear suppliers to receive the discount. Advice provided to participants about footwear is included in Online Resource 1. All other treatment, such as skin or nail care, exercises, or other aids, will be provided in line with standard podiatry practice in Australia.

The control group will receive usual care plus a self-management package based on advice from Creaky Joints Australia (29 Tips from Podiatrists for Managing Arthritis Pain in Your Feet), which provides a range of education including regular self-inspection of feet, advice about exercising to reduce foot/ankle discomfort, selecting appropriate footwear, and the use of supportive aids such as foot orthoses, ice packs, salt baths etc. For usual care, participants will be advised they can seek any treatment they prefer over the 12-week duration of the trial and their progress will be monitored. At the conclusion of the trial, participants randomised to the control group will be invited to attend a debriefing session and will be offered the same treatment as the intervention group if the intervention is found to be effective.

### Patient and public involvement

Recognising the importance of designing patient-centred research, we included a consumer as a coauthor (KL) during the initial design of the trial and securing grant funding. To further inform the trial design, we conducted four semi-structured interviews with people who have a rheumatological condition and foot-specific complaints. These interviews aimed to gather detailed feedback on various aspects of the trial, including the acceptability of the intervention, the feasibility of the proposed procedures, potential barriers to participation, and what outcomes are important. The interviews were conducted prior to the approval of the ethics application.

Participants will be recruited from outpatient rheumatology clinics at Austin Health in Melbourne, Australia. Rheumatologists will be informed about the trial and asked to recommend that their patients consider participating if they have foot-specific symptoms. Therefore, rheumatologists will initially approach participants about participating in the study and recommend that potential participants contact the researchers via a website. To be eligible for inclusion, participants must:

1. be adults over 18 years of age;
2. have a diagnosis of a rheumatological condition from a rheumatologist;
3. have foot-specific symptoms rated 3/10 or greater on a numerical rating scale (e.g. pain, stiffness, dermatological complaints); and
4. have experienced symptoms for at least four weeks.

Participants will be excluded if they:

1. are unable to understand the English language in verbal or written form;
2. have regularly attended a podiatry service for management of their rheumatological foot problem in the past 3 months;
3. have an active bacterial infection of the foot; and
4. experience foot symptoms that are not related to the rheumatological condition.

The recommended sample size for feasibility studies is 12 people per group [21], however to allow for a 20% drop-out rate, we will recruit 15 participants per group. This is similar to the median sample size of 30 for pilot trials documented in the United Kingdom Clinical Research Network database [22]. Given the low-risk nature of the interventions, we are not specifying interim analyses or guidelines for stopping the trial.

### Main outcome variable: feasibility

Given this is a pragmatic trial, the framework developed by Chan et al. [23] will be used to determine feasibility. According to this framework, 10 important domains of uncertainty should be addressed in a pragmatic feasibility trial. These domains are outlined in Table 1 along with criteria that will determine whether a larger trial is feasible. In addition to the feasibility outcomes, data will be collected on participant characteristics, adherence to interventions, adverse effects and acceptability and credibility of the interventions. These outcomes are outlined in detail in Online Resource 2.

**Table 1:** Domains and criteria used to evaluate trial feasibility

Domains of uncertainty	Criteria
1. Intervention development	No significant barriers to implementation of interventions are identified
2. Research ethics	No concerns are identified by the ethics committee
3. Participant identification and eligibility	There is a conversion rate of at least 75% (i.e. at least 75% of people screened are enrolled in the trial)
4. Recruitment	At least 5 participants are recruited per month
5. Setting	At least 60% of participants are recruited from rheumatology departments located in hospitals in northern Melbourne, Australia
6. Organisation	Resources required for the trial do not exceed the budget
7. Flexibility of delivery	There are minimal deviations from how interventions are delivered in clinical practice
8. Flexibility of adherence	The median self-reported adherence to the intervention is at least 3 ‘Sometimes’ on a Likert scale from 1, ‘Strongly disagree’ to 5 ‘Strongly agree’
9. Follow-up	Questionnaire completion rates at weeks 6 and 12 are at least 75%
10. Primary outcome	Semi-structured consumer interviews identify goal attainment as the most important outcome

### Other variables: limited efficacy testing

In addition to evaluating feasibility, limited efficacy testing will be undertaken on measures to be included in a fully powered randomised trial. These measures are outlined briefly in Table 2 and in specific detail in Online Resources 2.

**Table 2:** Other variables measured for limited efficacy testing

Variable	Outcome measure	Timepoints
Goal attainment	Goal Attainment Scaling	Baseline and week 12
Global change	Global Perceived Rating of Change (15-point Likert scale)	Week 6 and week 12
	Patient Global Assessment	Baseline, week 6 and week 12
Foot-specific pain	Foot Health Status Questionnaire—foot pain domain	Baseline, week 6 and week 12
	Manchester-Oxford Foot Questionnaire—foot pain subscale	Baseline, week 6 and week 12
Foot-specific function	Foot Health Status Questionnaire—foot function domain	Baseline, week 6 and week 12
	Manchester-Oxford Foot Questionnaire—walking/standing subscale	Baseline, week 6 and week 12
Disability	Health Assessment Questionnaire—disability index	Baseline, week 6 and week 12
Stiffness	11-point numerical rating scale	Baseline, week 6 and week 12
Fatigue	Functional Assessment of Chronic Illness Therapy-Fatigue (FACIT-Fatigue) version 4	Baseline, week 6 and week 12
Change in physical activity	Global Physical Activity Questionnaire	Baseline, week 6 and week 12

### Procedures

**Fig. 1:**
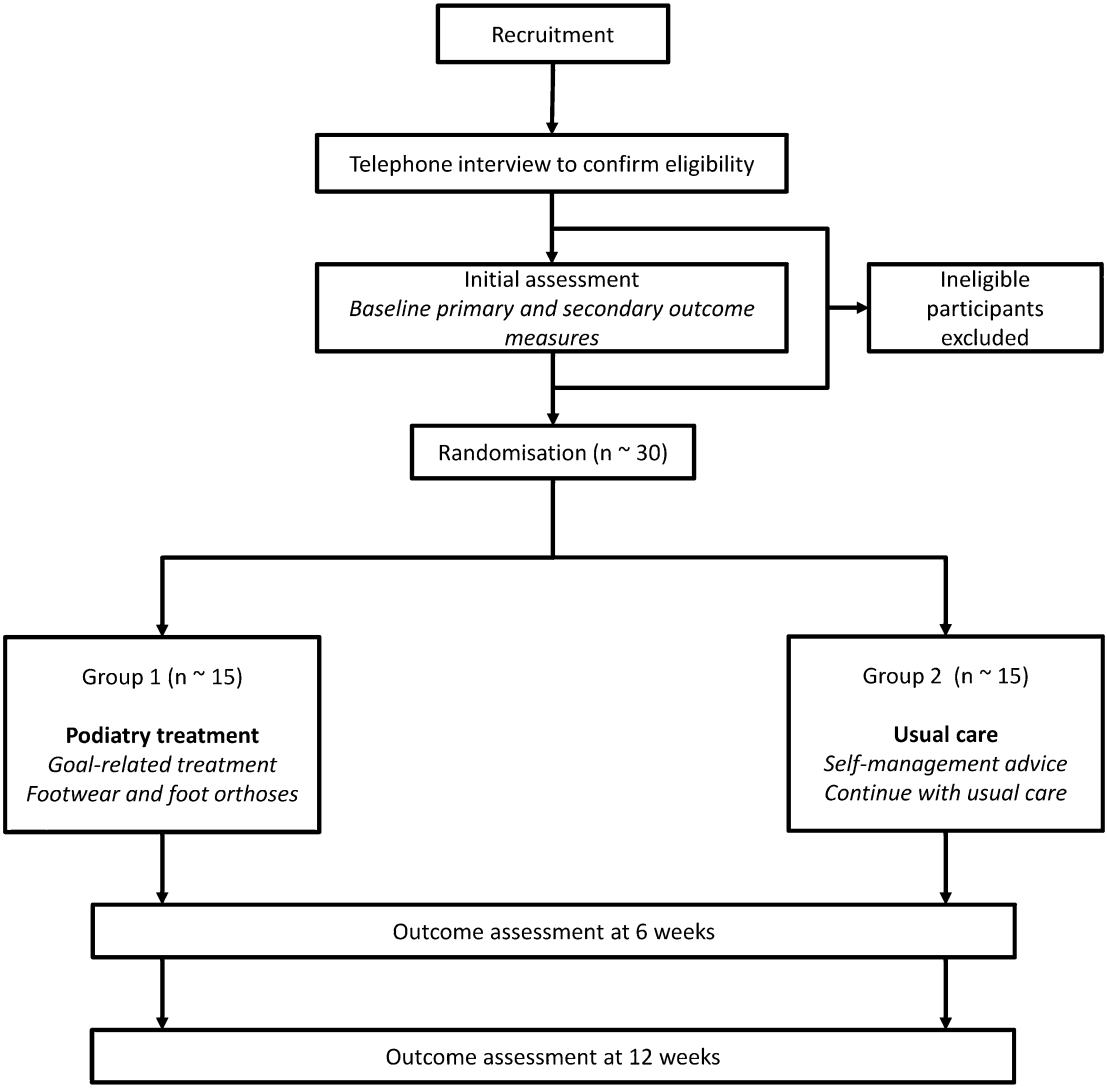
Diagram of participant flow

The flow of participants through the trial is displayed in Fig. 1. Potential participants will be initially screened via telephone interview against the inclusion criteria by a research assistant, who will enter data into REDCap. The data collected during this screening telephone interview is outlined in Online Resource 3. During eligibility screening, if the participant is eligible for inclusion an appointment will be made for the baseline assessment with the Chief Investigator. At the baseline appointment, the Chief Investigator will randomise participants to either the intervention or control group after obtaining informed consent to participate in the trial.

The total period of data collection for participants is 12 weeks. Participants who are enrolled in the trial will attend a baseline appointment at La Trobe University Health Sciences Clinic to complete outcome measures and commence podiatry treatment or receive the control intervention depending on their allocation. All outcomes will be completed on a computer and recorded in REDCap except for goals developed for Goal Attainment Scaling, which will be recorded on hard copy sheets for each individual participant. The change in Goal Attainment Scaling (rather than the goal itself) will be recorded by the Chief investigator in REDCap at the week 12 follow-up assessment. At week 6, the research assistant will send outcome measures to participants by email with a link to the REDCap survey. Follow-up assessment at week 12 will be in-person at the La Trobe University Health Sciences Clinic. Participants who are randomised to the intervention group will also attend treatment visits at the La Trobe University Health Sciences Clinic, which will occur depending on the treatment required.

### Statistical analysis

Descriptive statistics will be used to report feasibility outcomes. Mean (SD) scores and mean differences (95% CI) will be used to explore differences in continuous variables between the groups and Cohen’s *d* will be calculated to evaluate efficacy for the secondary outcome measures. A small effect size (Cohen’s *d* ≥ 0.20) will be regarded as an acceptable between group difference to signal efficacy. Data that is not continuous (adverse effects and use of cointerventions) will be reported using frequencies, medians and interquartile ranges as appropriate. Between group differences will be explored using risk ratios. Missing data will be minimised as much as possible, however no statistical approaches will be used to replace missing data. Any missing data will be evaluated and reported.

## Discussion

This study aims to assess the feasibility of conducting a pragmatic randomised trial to evaluate the effectiveness of a publicly-funded podiatry service for people with foot-related symptoms due to rheumatological conditions. The study will also explore the appropriateness of using patient-centred goal-setting as an outcome measure. People with rheumatological conditions often experience severe foot-related symptoms that significantly impact their daily lives. Despite advancements in treatment, these symptoms may persist, highlighting the need for ongoing multidisciplinary collaboration to improve patients’ health and wellbeing. There is a notable lack of podiatric care within multidisciplinary teams in Australia [14], which is crucial for optimal management of these foot-related issues.

One UK-based randomised trial has evaluated podiatry care for people with rheumatoid arthritis [24]. This exploratory trial randomised participants to receive podiatry-led foot care for 12 months or a no treatment control group. The authors reported several considerations for the design of future fully powered randomised trials that we have considered during the design of this trial. First, the findings suggest that podiatry-led foot care maintains but does not improve foot-related impairment or disability in patients with stable, controlled rheumatoid arthritis. These findings suggest that podiatry-led care is important for maintaining foot health. A consideration for these findings was the use of a standardised outcome measure (the Leeds Foot Impact Scale) that may not be responsive to change [25]. Therefore, selecting an outcome measure that is both meaningful for patients and sensitive and responsive is important. Our feasibility trial includes multiple patient-centred outcome measures to allow evaluation of their performance for a larger randomised trial. Second, the trial faced difficulties recruiting participants due to stringent criteria for stable drug management and co-morbid diseases such as diabetes and peripheral vascular disease. The authors reported the criteria were too strict and therefore excluded many potential participants. For our feasibility trial, the inclusion criteria are broader and include any rheumatological condition without excluding participants with comorbidities. Third, more than one-third of patients refused to participate in the trial, mainly due to additional clinic visits, lack of understanding of podiatry care, and concerns about the non-intervention arm. Clear communication about the benefits and risks of podiatry care is therefore essential.

One objective for this feasibility trial is to evaluate the feasibility of using patient-centred goal attainment outcome measures (Goal Attainment Scaling). Goal Attainment Scaling is a structured method used to set and measure the achievement of patient-centred goals. The overall effectiveness of the intervention is determined by aggregating individual goal scores into a single t-score. Goal Attainment Scaling has several benefits, such as allowing patient-centred outcomes, greater sensitivity to change compared to standardised measures, and flexibility in settings with heterogenous participants. However, there are some challenges when implementing Goal Attainment Scaling in a randomised trial, such as ensuring consistent goal-setting across different practitioners, managing patient expectations, and dealing with the subjective nature of goal assessment [26].

This protocol outlines the methods for a participant-blinded, parallel-group randomised feasibility trial to compare the addition of a podiatry service to usual care for people with a rheumatological condition and foot specific symptoms. In addition to determining whether a larger randomised trial is feasible, this trial will also evaluate whether it is feasible to use Goal Attainment Scaling as a primary outcome measure and provide data to determine a suitable sample size.

## Declarations

### Data availability

Data are available from the authors upon reasonable request.
